# Colonic polyps: a rare clinical manifestation of schistosomiasis (a case report)

**DOI:** 10.11604/pamj.2022.42.187.34227

**Published:** 2022-07-07

**Authors:** Kelvin Orare, Abdinoor Mohamed, Monali Thakkar, Allan Rajula, Joseph Gatheru

**Affiliations:** 1 Department of Medicine, Faculty of Health Sciences, Aga Khan University Medical College, Nairobi, Kenya

**Keywords:** Colonic polyps, schistosomiasis, neglected, case report

## Abstract

Schistosomiasis is caused by parasitic blood flukes. It is one of the neglected tropical diseases (NTDs) and has variable manifestations depending on the species involved. Eggs and not adult worms are mostly responsible for the resultant pathologies commonly involving the small intestines. Colon polyps resulting from schistosomiasis represent a rare entity despite the endemicity of Schistosoma mansoni in the tropical regions. Praziquantel is the mainstay of treatment. Case presentation is of a 28 year old male with complaints of bloating, mild abdominal pain and loose stools over two weeks.On examination, the patient´s abdomen was soft on palpation with a liver span of 8 cm and a non-palpable spleen. Preliminary tests included stool microscopy which was negative for ova and cysts, negative for stool H. pylori antigen. Colonoscopy revealed a polyp at the caecum and pan-colonic erosions. Microscopy of the polyp showed numerous schistosome ova. The indolent picture of this particular patient makes it a unique case as the likelihood of the diagnosis not being schistosomiasis is greater than other differential diagnoses. The take-home message is that a high index of suspicion is needed in the diagnosis of colonic polyps resulting from schistosomiasis as it presents with non-specific manifestations and inconclusive laboratory and endoscopic findings.

## Introduction

Schistosomiasis (Bilharziasis) is a disease caused by trematodes from the family Platyhelminthes and the genus schistosomatidae [1]. It is one of the neglected tropical diseases (NTDs) and has variable manifestations depending on the species involved. At-risk populations mainly consist of school-age children in environs of poor sanitation and infected water containing infectious cercariae [2]. Colon polyps resulting from schistosomiasis represent a rare entity despite the endemicity of *Schistosoma mansoni* in the tropical regions and common intestinal involvement. The disease affects more than 200 million people worldwide [3]. The following is a case of a colonic polyp associated with schistosomiasis.

## Patient and observation

**Patient information**: we present the case of a 28-year-old male who presented to the gastroenterology outpatient clinic with complaints of bloating, mild abdominal pains, and loose stools over two weeks. The stools were soft, non-bloody, non-mucoid occurring at a frequency of two to three times a day, mostly post-prandial. The frequency of bowel motions was worsened by having spicy foods. The loose bowel motions were associated with right lower abdominal pain, bloating, and borborygmi. There was no history of nausea, vomiting, or pyrosis. On further query, the patient reported no fevers, night sweats, chills, and difficulty in breathing or weight loss. He also denies any dysuria, frequency, hematuria, and incontinence. There was no evidence of immunosuppression. His past medical history was unremarkable. He had had no previous admissions, no surgeries, and was not on treatment for any chronic illness. He was allergic to red meat. He did not smoke or drink alcohol. The patient was on self-prescribed metronidazole during the two weeks before presentation to the clinic with no improvement of symptoms. He worked as a clerk in the Nairobi City county administration office. During his childhood years, his family lived in Homa Bay town near Lake Victoria where he and his brothers often took baths.

**Clinical findings**: on examination findings, he had no pallor or jaundice. He had a blood pressure of 139/94 mmHg, a temperature of 36.7 celsius, respiratory rate of 20/min, a pulse at 63 per minute, and saturating at 98% on ambient room air. His abdomen was soft on palpation, the liver span was 8 cm and the spleen was not palpable. His lower limbs appeared normal with no evidence of edema. There were no neurological deficits.

**Diagnostic assessment**: preliminary tests included stool microscopy which was negative for ova and cysts, negative stool *H. pylori* antigen, normal hemogram including differential count, and a normal urinalysis. An ultrasound of the liver and gallbladder was suggestive of hepatic steatosis grade I.

**Figure 1:**
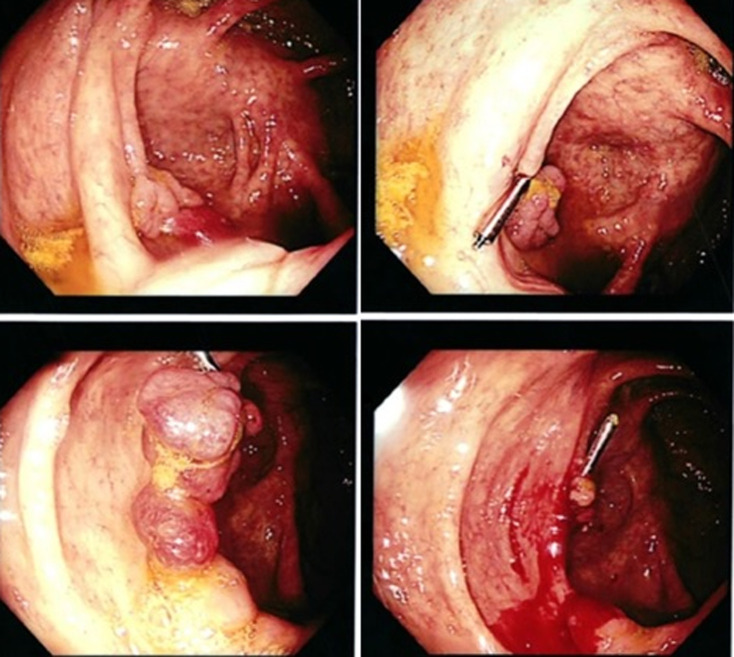
colonoscopy images showing polyp at the caecum and pan-colonic erosions

**Figure 2:**
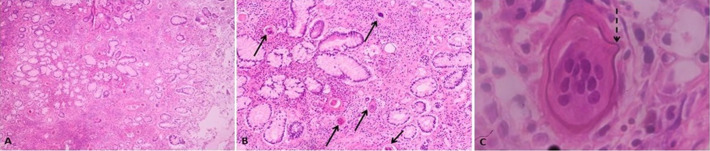
microscopic examination of the polyp showing extensive eosinophilic inflammation in the lamina propria along with numerous schistosome ova (A,B,C)

**Therapeutic interventions**: further evaluation included a colonoscopy which revealed a polyp at the caecum and pan-colonic erosions (Figure 1). The polyp was resected and random colonic biopsies were taken and submitted for histopathology evaluation. Microscopic examination of the polyp showed extensive eosinophilic inflammation in the lamina propria along with numerous schistosome ova (Figure 2). Sections from the random colon biopsies showed chronic colitis with eosinophilia. The patient was then put on praziquantel 1200mg single dose.

**Informed consent**: informed consent was sought and obtained from the patient. Anonymity was maintained for confidentiality.

**Patient perspective**: when we first met the patient in the outpatient clinic, he was already taking metronidazole and hadn´t felt any relief in his symptoms. We then transitioned him to praziquantel which resulted in clinical improvement, to his relief. A follow up with him 1 year later revealed no symptom recurrence.

## Discussion

Gastrointestinal disease resulting from schistosomiasis is caused by two major species i.e., *Schistosoma mansoni and S. japonicum. S. mansoni* is the predominant species causing disease in the East African region [3]. The highest prevalence is usually seen along with the Lake Victoria, and surrounding islands- Kano Plain (Bondo, Kisumu West, Kisumu East, Nyando, Suba, Rachuonyo, and Homa Bay in Nyanza Province) and areas along the coastal strip [4]. In our case, the patient hails from an endemic area-Homa Bay county, near Lake Victoria where he could have been exposed. The disease predominantly affects those in the first two decades of their lives. Most patients will be asymptomatic during the acute phase. Some, however, develop acute schistosomiasis syndrome (Katayama fever), particularly those from non-endemic areas. Chronic established infection is theorized to occur due to entrapment of schistosome eggs during migration.

Infection occurs after a person comes in direct contact with the cercaria [5]. The definitive hosts of the disease are humans, where sexual reproduction and maturation occur. Freshwater snails (*Biomphalaria spp*) are the intermediate host for *S. mansoni*, where the cercaria develop [6]. There is a wide presentation of symptoms in intestinal schistosomal disease- from asymptomatic presentation to vague abdominal pain, change in bowel movements (commonly diarrhea), and/or blood in the stool [7]. Liver involvement causing abdominal distention, jaundice, ascites, or portal hypertension may also be noted [8].

Polyp formation can occur due to intestinal wall implantation of viable eggs containing miracidia. This causes an acute local inflammatory reaction- leading to congestion and edema and further forming granulomas. These can ulcerate and bleed, causing hematochezia or melena stools. This granuloma formation can further complicate the diagnosis of Schistosoma polyps if there is no recent encounter with lakes or water bodies, thus putting schistosomiasis as a differential at a lower level. Stool examination for schistosome ova and colonoscopy remains the gold standard of diagnosis. Ova are passed frequently in stool only in the early stages with the frequency decreasing as the disease takes on a chronic course. Other direct assays include the demonstration of schistosome antigen in blood, urine, or stool. Antigen tests use circulating anodic antigen or circulating cathodic antigen detected in urine or blood [9]. Indirect assays such as detection of antibodies can be done particularly in low-endemic areas, and people with low parasitic burden [9]. Endoscopic findings in colonic schistosomiasis remain non-specific, therefore the clinical and pathological correlation is paramount [10].

## Conclusion

This case report highlights the rare manifestations of colonic schistosomiasis in a patient with no comorbid disease who grew up in Homa Bay, a town near Lake Victoria where schistosomiasis is a known endemic disease but who traveled to Nairobi over 10 years ago where manifestations of schistosomiasis are minimal. The diagnosis for such a disease in its latent/chronic stage is usually challenging given the false-negative rates for stool examination are often high at this stage of the disease. Hence, a high index of suspicion for schistosomiasis needs to be in place for any patient presenting with vague intestinal symptoms that have a history of travel or residence in an area that has a freshwater body.
